# Screening and Evaluation of *Lactiplantibacillus plantarum* HNGD-YC1 for Mitigation of Advanced Glycation End Products in Food Matrices

**DOI:** 10.3390/foods15193403

**Published:** 2026-09-24

**Authors:** Yueting Ge, Kaile Chen, Zhenguo Tian, Chengjia Gu, Boyang Kou, Guowei Le, Yuhui Yang

**Affiliations:** 1 Henan Key Laboratory of Tea Plant Biology, College of Tea and Food Science, Xinyang Normal University, Xinyang 464000, China; ytge@xynu.edu.cn (Y.G.);; 2 Dabie Mountain Laboratory, Xinyang 464000, China; 3 Henan International Joint Laboratory of Tea-Oil tree Biology and High-Value Utilization, College of Tea and Food Science, Xinyang Normal University, Xinyang 464000, China; 4 College of Food Science and Engineering, Henan University of Technology, 100 Lotus Street, Hi-Tech Development District, Zhengzhou 450001, China; 5 State Key Laboratory of Food Science and Technology, School of Food Science and Technology, Jiangnan University, Wuxi 214122, China

**Keywords:** advanced glycation end products, lactic acid bacteria, *Lactiplantibacillus plantarum*, screening, identification

## Abstract

Advanced glycation end products (AGEs), stable glycation/glycoxidation products generated during thermal processing, accumulate in foods and pose health risks. Current mitigation strategies primarily focus on inhibiting AGEs formation rather than reducing already formed products, highlighting the need for effective approaches to reduce pre-formed AGEs. In this study, a food-grade lactic acid bacterium (LAB) capable of efficiently reducing fluorescent AGEs was isolated from naturally fermented foods using AGEs as the primary nitrogen source. From 1213 presumptive LAB isolates, three candidates with fluorescent AGEs reduction rates exceeding 50% were obtained, among which strain HNGD-YC1 exhibited the highest activity and was selected for further characterization. The strain was identified as *Lactiplantibacillus plantarum* through morphological, phylogenetic, and whole-genome ANI analyses and demonstrated favorable in vitro probiotic-related properties, including acid and bile salt tolerance, as well as a genomic safety profile. Whole-genome sequencing revealed candidate genes encoding proteases, glycoside hydrolases, and carbohydrate esterases, suggesting a putative multi-enzymatic mechanism involved in the reduction of fluorescent AGEs, which warrants further experimental validation. In food matrix applications, fermentation with HNGD-YC1 reduced fluorescent AGEs by 68.70% in spray-dried milk powder and 56.39% in roasted pork powder, while also reducing multiple other protein oxidation products and partially recovering the protein secondary structure parameters. These findings establish HNGD-YC1 as a promising candidate, providing a novel approach for developing low-AGEs fermented products.

## 1. Introduction

Protein oxidation is a ubiquitous chemical reaction occurring during food processing and storage, involving the covalent modification of protein molecules by reactive oxygen species and other free radicals [1,2]. The primary mechanisms include oxidative modification of amino acid side chains, cleavage of the protein backbone, and formation of intermolecular and intramolecular covalent crosslinks [3,4]. These reactions irreversibly alter protein conformation and physicochemical properties, leading to food quality deterioration, nutritional loss, and potential health risks [5,6]. In modern food processing, protein oxidation is prevalent across the entire supply chain from raw material handling to processing and storage [7,8]. For instance, spray-drying in the dairy industry (inlet temperatures often exceeding 160 °C) induces oxidative modifications of whey proteins and casein [9], while high-temperature roasting and frying are major drivers of myofibrillar protein oxidation in meat products [10]. Therefore, protein oxidation has become a common challenge affecting food quality and nutritional safety across the food industry.

Advanced glycation end products (AGEs) are stable compounds formed primarily through non-enzymatic glycation reactions between reducing sugars and the free amino groups of proteins, such as the ε-amino group of lysine, via the Maillard reaction during thermal processing [11,12]. Glycation and protein oxidation are often closely coupled, and AGEs are therefore frequently measured together with other oxidation-associated markers [13]. They are widely present in heat-processed foods such as baked and roasted products, and their content is particularly elevated in thermally processed dairy and meat products [14]. Once formed, AGEs are chemically stable and can accumulate continuously during food processing and storage without being degraded under subsequent mild treatments [15,16]. AGEs disrupt protein structure and function, leading to reduced nutritional value of foods [15]. More critically, dietary intake of AGEs contributes significantly to their accumulation in the human body, and long-term consumption of high-AGEs diets has been consistently associated with increased risks of various chronic metabolic diseases, including type 2 diabetes, cardiovascular diseases, neurodegenerative disorders, and inflammatory bowel diseases [17,18]. Mechanistically, AGEs induce oxidative stress and inflammatory responses [19,20], which are key drivers of these pathological conditions [21]. Therefore, developing safe and effective strategies to reduce AGEs in foods has become an important issue in the field of food nutrition and safety.

Current strategies for controlling AGEs in foods predominantly rely on three approaches: physical methods, chemical inhibitors, and enzymatic degradation. Physical methods such as low-temperature storage and vacuum packaging can reduce the generation of free radicals but have no effect on already formed stable products. Chemical methods, including the addition of exogenous antioxidants [22,23], pose risks of reagent residues and safety concerns [15]. Enzymatic methods, despite their specificity, are limited by high costs and poor storage stability, making large-scale industrial application difficult. Moreover, AGEs are inherently poorly degradable compounds [24], and the crosslinks they form can impair proteolytic digestibility [25], further complicating enzymatic approaches. In summary, the current technological system for controlling AGEs in foods suffers from a fundamental limitation: heavy emphasis on preventing the formation of AGEs while neglecting the reduction of those already formed and accumulated in processed foods. There remains a lack of safe, efficient, and scalable strategies for reducing pre-existing AGEs in food matrices, as most current research has focused on inhibiting AGEs formation rather than reducing already formed products [15].

In this context, lactic acid bacteria (LAB), many of which are generally recognized as safe (GRAS) and are widely used in fermented foods, have emerged as promising candidates for the biotransformation and removal of food-borne hazardous compounds [26]. LAB possess the ability to utilize sugars and proteins for growth, and given that AGEs are essentially products of non-enzymatic reactions between sugars and proteins, the substrate utilization compatibility provides a theoretical basis for LAB to reduce AGEs. Previous studies have demonstrated that LAB can reduce various food contaminants, including acrylamide, organophosphorus pesticides, purine nucleosides, branched-chain amino acids, and other potentially harmful compounds, through enzymatic hydrolysis, physical adsorption, and metabolic transformation [27,28,29,30,31,32]. Regarding AGEs specifically, recent research has revealed that certain LAB strains can inhibit AGEs formation during food fermentation. Li et al. found that *Lactobacillus fermentum* significantly inhibited the formation of total fluorescent AGEs and carboxymethyl lysine (CML)/carboxyethyl lysine (CEL) during vinegar fermentation [33]. Yue et al. reported that *Lactiplantibacillus plantarum* CCFM 1354 could inhibit AGEs formation and alleviate skin aging, though the study focused on a peanut skin scenario without addressing the reduction of pre-formed AGEs in foods [34]. Kumar et al. screened LAB from human milk with anti-glycation activity, but their work remained at the in vitro stage without food matrix application studies [35]. However, the vast majority of existing studies focus on inhibiting the formation of AGEs rather than reducing already formed AGEs. Research on the direct reduction of pre-existing AGEs in complex food matrices by LAB remains limited, and to the best of our knowledge, no study has systematically investigated this process coupled with genomic elucidation of the underlying mechanisms.

Therefore, the present study aimed to screen food-grade LAB from naturally fermented foods collected across multiple provinces in China that are capable of efficiently reducing fluorescent AGEs, using AGEs as the primary nitrogen source in a selective medium. The objectives of this study were to evaluate the in vitro probiotic-related properties and safety of the selected strain, investigate its efficacy in reducing fluorescent AGEs in real food matrices (spray-dried milk powder and roasted pork powder), and elucidate the putative molecular mechanisms through whole-genome sequencing and functional annotation. The findings of this study are expected to provide a novel green strategy for the biological mitigation of fluorescent AGEs and other oxidation-associated markers in foods and to offer a theoretical basis for the development of potential probiotic products capable of reducing these markers in food matrices.

## 2. Materials and Methods

### 2.1. Sample and Reagent Preparation

A total of 370 naturally fermented food samples were collected from various provinces across China, covering eight major categories including fermented bean, vegetable, meat, flour, dairy, fruit, wine, and tea products. All samples were stored at 4 °C prior to use. Pasteurized milk and fresh pork tenderloin were purchased from a local supermarket (Zhengzhou, China). For milk powder preparation, pasteurized milk was freeze-dried as the low-oxidation control, while pasteurized milk subjected to spray-drying (180 ± 5 °C inlet, 90 ± 5 °C outlet, 0.2 MPa) served as the high-oxidation group. For pork powder, fresh pork tenderloin was sliced (5 mm), vacuum-sealed, cooked at 65 °C for 10 h, freeze-dried, pulverized, and sieved to obtain the low-oxidation control (Sous-vide cooked). The high-oxidation group was prepared by roasting at 180 °C for 30 min, followed by the same processing steps. All samples were stored at −80 °C. AGEs were prepared by dissolving 14.6 g lysine, 50.0 g bovine serum albumin (Shanghai Yuanye Bio-Technology Co., Ltd., Shanghai, China), and 100.0 g glucose in distilled water (1 L), incubated at 50 °C for 4 days under aseptic conditions [36], filter-sterilized (0.22 μm), and stored at −20 °C. Phosphate-buffered saline (PBS, pH 7.2) was prepared by dissolving 3.58 g of Na_2_HPO_4_·12H_2_O, 0.24 g of KH_2_PO_4_, 8.00 g of NaCl, and 0.20 g of KCl in distilled water to a final volume of 1 L. All media and buffers were autoclaved at 121 °C for 20 min. All chemicals were of analytical grade.

### 2.2. Isolation and Screening of LAB for Reduction of Fluorescent AGEs

LAB isolation and purification were performed according to the method described by Liu et al. [32] with minor modifications. Briefly, samples were serially diluted with sterile saline and spread onto Man–Rogosa–Sharpe (MRS) agar plates supplemented with 0.04% (*w*/*v*) bromocresol purple, then incubated at 37 °C for 48 h. Colonies producing yellow clear zones were selected and purified by repeated streaking on fresh MRS agar plates. The purified isolates were then subjected to Gram staining and catalase testing. Strains that were Gram-positive and catalase-negative were selected for subsequent determination of their ability to reduce fluorescent AGEs.

A two-step screening strategy was employed. For primary screening, a selective medium containing AGEs as the primary nitrogen source was used. The medium contained CH3COONa (5.0 g/L), glucose (20 g/L), K_2_HPO_4_ (2.0 g/L), ammonium citrate dibasic (2.0 g/L), MgSO_4_·7H_2_O (0.2 g/L), MnSO_4_·H_2_O (0.04 g/L), Tween 80 (1.0 mL/L), and AGEs solution (10 mL/L). Ammonium citrate dibasic was included as a basal nitrogen source in the selective medium to support initial bacterial growth, while AGEs served as the primary target nitrogenous substrate for screening. Each isolate was activated in MRS broth at 37 °C for 24 h. After incubation, the activated cultures were centrifuged, washed twice with sterile saline, and their optical density at 600 nm (OD_600_) was adjusted to 1.0 with sterile saline. The washed strains were inoculated (2%, *v*/*v*) into fresh selective medium and incubated at 37 °C for 72 h. After fermentation, the cultures were centrifuged and the supernatants were collected. AGEs-related fluorescence intensity was determined at an excitation wavelength of 370 nm and an emission wavelength of 440 nm using a multifunctional microplate reader (Varioskan LUX, Thermo Fisher Scientific, Waltham, MA, USA), according to the fluorimetric method for fluorescent AGEs summarized by Perrone et al. [37]. The uninoculated selective medium was used as the blank control (F_0_), and the supernatant of the inoculated medium was recorded as F_1_. The reduction rate of fluorescent AGEs was calculated as: (F_0_ − F_1_)/F_0_ × 100%. Because fluorescent AGEs represent only a subset of total AGEs and fluorescence signals may be influenced by other fluorophores, this value is reported as the reduction in fluorescent AGEs rather than a validated measure of total AGE chemical degradation.

Strains with reduction rates exceeding 50% in the primary screening were selected as candidates for secondary screening. The candidate strains were activated in MRS broth at 37 °C until their optical density at 600 nm (OD600) reached 1.0. Subsequently, the cultures were inoculated (2%, *v*/*v*) into fresh MRS broth supplemented with AGEs solution (10 mL/L) and incubated at 37 °C for 24 h. After incubation, the cultures were centrifuged and the supernatants were collected. Uninoculated MRS broth supplemented with AGEs was used as the background control. The fluorescence intensity of fluorescent AGEs in the supernatant was determined using the same method as described for the primary screening [37], and the reduction rate was calculated accordingly. The strain with the highest and most stable AGEs-reducing activity was ultimately selected for subsequent characterization and application studies.

### 2.3. In Vitro Probiotic-Related Properties and Safety Evaluation

Acid and bile salt tolerance were evaluated according to Gupta et al. [38]. Artificial gastric juice was prepared by dissolving pepsin (0.9 g/L, Sigma-Aldrich, St. Louis, MO, USA) in sterile PBS, adjusted to pH 2.0 and 3.0. Artificial intestinal juice was prepared with trypsin (3.359 g/L, Sigma-Aldrich, USA) and 0.15% or 0.3% (*w*/*v*) ox bile salt (Shanghai Aladdin Bio-Chem Technology Co., Ltd., Shanghai, China) in PBS, pH 7.5. All solutions were filter-sterilized through 0.22 μm membranes prior to use. Candidate strains were activated in MRS broth at 37 °C for 18 h, harvested, washed with PBS, and resuspended to ~10^8^ CFU/mL. Aliquots of the cell suspension (1 mL) were inoculated into 9 mL of the artificial gastric or intestinal juice and incubated at 37 °C. Samples were taken at 0, 2, and 4 h, serially diluted with PBS, and viable cell counts were determined by plating on MRS agar plates. The survival rate was calculated as: Survival rate (%) = (A_t_/A_0_) × 100, where A_t_ is the viable cell count after 2 or 4 h of incubation, and A_0_ is the initial viable cell count at 0 h.

Cell surface hydrophobicity and auto-aggregation capacity were measured according to previously described methods [39,40]. For hydrophobicity, activated cultures were harvested, washed twice with PBS, and resuspended to an optical density of approximately 0.5 at OD_600_ (A_0_). An equal volume of xylene was added and mixed by vortexing for 2 min. After incubation at 37 °C for 1 h to allow phase separation, the aqueous phase was carefully collected, and its OD_600_ (A_1_) was measured. The hydrophobicity was calculated as: Hydrophobicity (%) = [(A_0_ − A_1_)/A_0_] × 100. For auto-aggregation, the cell suspension was adjusted to an OD_600_ of approximately 0.5 (OD_0_) and incubated at 37 °C without shaking. The OD_600_ of the upper phase was measured at 4 h (OD_t_). The auto-aggregation capacity was calculated as: Auto-aggregation (%) = [1 − (OD_t_/OD_0_)] × 100.

Hemolytic activity was assessed on Columbia blood agar plates according to the method described by Ou et al. [41]. The activated strain HNGD-YC1 (~10^8^ CFU/mL) was streaked onto Columbia blood agar plates containing 5% defibrinated sheep blood and incubated at 37 °C for 48 h under anaerobic conditions. *Staphylococcus aureus* ATCC 25923 was used as a positive control (β-hemolysis), and *Lactiplantibacillus plantarum* ATCC 14917 was used as a negative control (γ-hemolysis). After incubation, the plates were examined for the presence of clear zones around the colonies, indicating β-hemolytic activity.

### 2.4. Identification and Growth Characterization

Genomic DNA of the selected strain was extracted using the Ezup Column Kit (Sangon Biotech Co., Ltd., Shanghai, China). The 16S rDNA gene was amplified by PCR using the universal bacterial primers 27F (5′-AGAGTTTGATCCTGGCTCAG-3′) and 1492R (5′-GGTTACCTTGTTACGACTT-3′). PCR products were sequenced by Sangon Biotech Co., Ltd. (Shanghai, China). Sequences were compared using BLAST+ version 2.16.0 against the NCBI database, and a phylogenetic tree was constructed using MEGA 7.0 software with the neighbor-joining method (1000 bootstrap replicates). For further species-level confirmation, ANI analysis was performed using the EzBioCloud ANI Calculator (OrthoANIu algorithm) by comparing the HNGD-YC1 genome with the type strain *Lactiplantibacillus plantarum* ATCC 14917ᵀ (NCBI RefSeq assembly accession: GCF_001434175.1).

The selected strain was streaked onto MRS agar plates and incubated at 37 °C for 24 h, after which the colony morphology (color, shape, size, texture, and edge) was observed. Gram staining was performed according to the method described by Wang et al. [42], and the stained cells were examined under an optical microscope (CX31 RTSF, Olympus Corporation, Tokyo, Japan). For ultrastructural observation, the strain was cultured in MRS broth until the mid-exponential phase, harvested by centrifugation, and fixed with 2.5% glutaraldehyde at 4 °C. After washing with PBS, the cells were dehydrated through a graded ethanol series, freeze-dried, coated with gold, and examined under a scanning electron microscope (Zeiss Sigma 300, Carl Zeiss Microscopy GmbH, Jena, Germany), according to the method described by Liu et al. [32].

To determine the optimal growth temperature, the activated strain was inoculated (2%, *v*/*v*) into MRS broth (pH 6.5) and incubated at different temperatures (27 °C, 32 °C, 37 °C, and 42 °C) for 24 h, after which the OD_600_ was measured. To determine the optimal initial pH, the strain was cultured in MRS broth with initial pH values adjusted to 5.0, 5.5, 6.0, 6.5, 7.0, 7.5, 8.0, and 8.5 at 37 °C for 24 h, followed by OD_600_ measurement. For growth and acid production curve analysis, the activated strain was inoculated (2%, *v*/*v*) into MRS broth and incubated at 37 °C. The OD_600_ and pH of the culture medium were recorded every 2 h to construct the growth curve and pH change curve.

### 2.5. Whole-Genome Sequencing

Genomic DNA of strain HNGD-YC1 was submitted to Biomarker Biotechnology Co., Ltd. (Beijing, China) for whole-genome sequencing using the Illumina MiSeq platform with a paired-end library. The obtained raw reads were quality-filtered and assembled de novo using SPAdes (version 3.15.3). Assembly quality was evaluated using QUAST (version 5.2.0). Gene prediction was carried out using Prokka (version 1.14.6). Functional annotation was performed by searching against multiple public databases, including the NCBI Non-Redundant Protein (NR), Clusters of Orthologous Groups (COG), eggNOG (Evolutionary Genealogy of Genes: Non-supervised Orthologous Groups), Gene Ontology (GO), and Kyoto Encyclopedia of Genes and Genomes (KEGG) databases. Carbohydrate-active enzymes (CAZymes) were annotated using the CAZyme database (dbCAN). Antibiotic resistance genes were identified using the Comprehensive Antibiotic Resistance Database (CARD) with the Resistance Gene Identifier (RGI). The default criteria were applied: Perfect hits required 100% identity and 100% coverage; Strict hits required a bit-score above the curated BLASTP cutoff for each ARG family; all remaining hits were classified as Loose hits. Potential virulence-related genes were screened against the Pathogen–Host Interaction database (PHI-base) using BLASTP with an E-value threshold of ≤ 1 × 10^−5^, a minimum identity of 30%, and a minimum coverage of 50%.

### 2.6. Evaluation of Reduction Efficacy of the Selected Strain in Food Matrices

Microbial fermentation has been widely employed to modify the nutritional composition and functional properties of food substrates [43]. Spray-dried milk powder and roasted pork powder (prepared as in Section 2.1) were reconstituted with distilled water at ratios of 1:9 and 1:4 (*w*/*v*), respectively. The activated HNGD-YC1 culture (10^8^ CFU/mL) was inoculated at 2% (*v*/*v*) and incubated at 37 °C for 48 h. Fermented samples were freeze-dried and stored at −80 °C. Uninoculated samples served as oxidation controls. For food matrices, the unfermented oxidized matrix was used as the matrix-matched reference for calculating the reduction in fluorescent AGEs. A panel of oxidation- and glycation-associated markers [44,45], including AGEs, carbonyls, Schiff bases, kynurenine, N′-formylkynurenine, free thiols, and free amino groups, was measured in fermented and control samples.

Kynurenine and N′-formylkynurenine were determined by fluorescence spectroscopy (Ex/Em: 365/480 nm and 325/434 nm, respectively) [46], expressed as A.U./g pro. Carbonyl content was determined by the DNPH colorimetric method [47,48], calculated using a molar extinction coefficient of 22,000 M^−1^·cm^−1^ and expressed as nmol/mg pro. Schiff bases were quantified by fluorescence spectroscopy (Ex/Em: 350/420 nm) [49], and results were expressed as A.U./g pro. Free thiols were measured with Ellman’s reagent (ε = 13,600 M^−1^·cm^−1^) and results were expressed as μmol/g pro [50]. Free amino groups were determined by the OPA spectrophotometric method [51] using a five-point serine standard curve (50–200 mg/L, R^2^ > 0.99). Protein content was measured by the Kjeldahl method.

### 2.7. Microstructural and Structural Analysis

Confocal laser scanning microscopy (CLSM) observation was performed as described previously [44]. Freeze-dried samples were reconstituted, and 1 mL of sample was mixed with 10 μL fluorescein isothiocyanate (FITC) (0.1%, *w*/*v*), incubated in the dark for 30 min, and observed under a confocal laser scanning microscope (OLYMPUS FV3000, Olympus Corporation, Tokyo, Japan) with an excitation wavelength of 488 nm. Fluorescence images were captured to evaluate protein aggregation.

Fourier-transform infrared (FTIR) spectroscopy was performed according to Sun et al. [52]. Freeze-dried samples were ground with KBr (1:100, *w*/*w*) and pressed into transparent pellets. Spectra were recorded over the wavenumber range of 4000–400 cm^−1^ (32 scans, 4 cm^−1^ resolution) using an ALPHA FTIR spectrometer (Bruker Optics GmbH, Ettlingen, Germany). Spectra were baseline-corrected and analyzed with OMNIC 8.2 and Peak Fit 4.0. Secondary structure components (α-helix, β-sheet, β-turn, random coil) were calculated from the amide I region (1700–1600 cm^−1^).

### 2.8. Statistical Analysis

All experiments were performed with three independent biological replicates (*n* = 3). Data were analyzed using SPSS 27.0 (IBM Corporation, Armonk, NY, USA) and expressed as mean ± standard error of the mean (SEM). Normality of the data and homogeneity of variances were assessed by Shapiro–Wilk and Levene’s tests, respectively. Group comparisons were performed by one-way analysis of variance (ANOVA) followed by Tukey’s HSD post-hoc test. For the gastrointestinal tolerance assays, each condition was analyzed independently using one-way ANOVA. For data with unequal variances, Tamhane’s T2 test was used. Significance was set at *p* < 0.05. Graphs were generated using GraphPad Prism 8.0.2 (GraphPad Software, San Diego, CA, USA). Different superscript letters indicate significant differences among groups (*p* < 0.05).

## 3. Results

### 3.1. Screening of LAB for Reduction of Fluorescent AGEs from Traditional Fermented Foods

From the 370 fermented food samples collected across China (Figure 1A), a total of 1213 isolates producing yellow clear zones were preliminarily obtained on MRS agar supplemented with bromocresol purple (Figure 1B). The AGEs-reducing capabilities of all isolates were initially evaluated using a selective liquid medium containing AGEs. As illustrated in Figure 1C, while the majority of the isolates displayed detectable AGEs-reducing activity across a wide range of reduction rate intervals, only three strains, designated as HNGD-YC1, HNGD-KL5, and HNGD-WY4, exhibited reduction rates exceeding the 50% threshold. Further quantification in the selective medium (Figure 1D) revealed that HNGD-YC1 exhibited the highest reduction rate (61.39%), followed by HNGD-KL5 (54.06%) and HNGD-WY4 (52.39%) (*p* < 0.05). To confirm this preliminary finding and further assess their reduction efficiency under more nutritionally complex conditions, the three candidate strains were subjected to a secondary verification in MRS broth supplemented with AGEs, which provides competing nitrogen sources to simulate the nutrient-rich environment of actual food matrices. When evaluated in this system (Figure 1E), HNGD-YC1 again demonstrated a significantly higher reduction rate (52.00%) compared to HNGD-KL5 (43.67%) and HNGD-WY4 (34.30%) (*p* < 0.05), confirming that its AGEs-reducing activity was maintained even in the presence of competing nitrogen sources. Collectively, these results identified HNGD-YC1 as the most potent AGEs-reducing candidate, warranting its selection for further characterization and application studies.

### 3.2. In Vitro Probiotic-Related Properties and Safety Evaluation of the Selected Strain

The survival of probiotic strains under gastrointestinal stress is a fundamental prerequisite for their functionality in vivo. To assess this, the acid and bile salt tolerance of the three candidate strains were systematically evaluated. As shown in Figure 2A, the survival rates of all strains decreased progressively with increasing acidity and prolonged incubation time. At pH 3.0, all three strains maintained survival rates above 70% after 2 h and above 65% after 4 h. However, when exposed to pH 2.0, a more substantial decline was observed, with survival rates dropping to approximately 20% after 4 h. Notably, strain HNGD-YC1 consistently exhibited significantly higher survival rates than the other two strains across all tested conditions (*p* < 0.05). In bile salt tolerance assays (Figure 2B), all strains displayed survival rates exceeding 70% after 2 h and above 60% after 4 h at 0.15% bile salts. At the higher concentration of 0.3% bile salts, the survival rates declined to approximately 45% after 2 h and 18% after 4 h, with HNGD-YC1 consistently exhibiting the highest survival rates among the three strains across all time points (*p* < 0.05). Beyond gastrointestinal survival, adhesion to the intestinal epithelium is considered an important property of potential probiotics, and it can be indirectly estimated by cell surface hydrophobicity and auto-aggregation capacity. As presented in Figure 2C, strain HNGD-YC1 exhibited the highest cell surface hydrophobicity (95.99%), significantly exceeding those of HNGD-KL5 and HNGD-WY4 (*p* < 0.05). A similar trend was observed for auto-aggregation capacity (Figure 2D), where HNGD-YC1 also displayed the highest rate (38.04%), substantially outperforming the other two strains (*p* < 0.05). The combination of robust stress tolerance and superior adhesive properties indicates that HNGD-YC1 possesses considerable adhesion potential in vitro. Based on this comprehensive evaluation, strain HNGD-YC1 was selected as the most promising candidate for further investigation.

To ensure the safety of HNGD-YC1 for food applications, its hemolytic activity was evaluated on Columbia blood agar plates. As illustrated in Figure 2E, the positive control (*Staphylococcus aureus* ATCC 25923) exhibited clear zones around the colonies, indicative of β-hemolysis. In contrast, neither the negative control (*Lactiplantibacillus plantarum* ATCC 14917) nor strain HNGD-YC1 produced any clear zones, showing γ-hemolysis. These results indicate that HNGD-YC1 does not produce hemolysin or exhibit hemolytic activity, suggesting a favorable safety profile for food applications.

### 3.3. Identification, Morphological, and Growth Characteristics of Strain HNGD-YC1

The 16S rDNA gene of strain HNGD-YC1 was sequenced for phylogenetic analysis. BLAST homology search against the NCBI database and subsequent phylogenetic tree construction (Figure 3A) placed HNGD-YC1 within the same clade as *Lactiplantibacillus plantarum* with 100% bootstrap support, strongly supporting its identification as *L. plantarum*. To further confirm species-level identification, ANI analysis was performed between HNGD-YC1 and the type strain *L. plantarum* ATCC 14917ᵀ. The ANI value was 99.11%, which is well above the 95% species threshold, confirming the species-level identification of HNGD-YC1 as *L. plantarum*. The morphological traits of strain HNGD-YC1 were examined through colony observation, Gram staining, and scanning electron microscopy. On MRS agar, the colonies appeared as circular, milky white, convex structures with smooth surfaces and neat edges (Figure 3B). Gram staining revealed that HNGD-YC1 was Gram-positive (Figure 3C), and scanning electron microscopy further confirmed its typical rod-shaped morphology (Figure 3D). To characterize the growth behavior of HNGD-YC1, the effects of temperature and initial pH on its growth were evaluated. As presented in Figure 3E,F, the strain exhibited optimal growth at 37 °C and initial pH 7.0. Under these optimal conditions, the growth curve followed a typical sigmoidal pattern, with the strain reaching the stationary phase after 12 h (Figure 3G). Concomitantly, the pH of the culture medium decreased during fermentation (Figure 3H), indicating strong acid-producing capacity. These results demonstrate that HNGD-YC1 possesses both rapid acidification capacity and remarkable acid tolerance.

### 3.4. Whole-Genome Sequencing Analysis

To investigate the genetic basis of the reduction of fluorescent AGEs in strain HNGD-YC1, whole-genome sequencing and functional annotation were performed. The genome consists of a single circular chromosome of 3,375,650 bp with a GC content of 44.43%, containing 4728 predicted protein-coding genes, 70 tRNA genes, and 16 rRNA genes (Figure 4A). Functional annotation was conducted using multiple public databases. eggNOG classification assigned 2610 genes (Figure 4B), revealing pronounced enrichment of genes associated with carbohydrate transport and metabolism (266 genes), energy production and conversion (120 genes), post-translational modification and protein turnover (61 genes), and signal transduction mechanisms (70 genes). GO analysis categorized 2479 genes into three main categories (Figure 4C), with genes involved in catalytic activity and metabolic processes being prominently represented. KEGG pathway analysis (Figure 4D) showed that genes were mainly enriched in amino acid metabolism, carbohydrate metabolism, and membrane transport pathways, with ABC transporters (109 genes) and biosynthesis of amino acids (107 genes) being among the most abundant categories. Additionally, pathways related to carbohydrate metabolism, including glycolysis/gluconeogenesis (52 genes), pyruvate metabolism (54 genes), and starch and sucrose metabolism (55 genes), were also enriched, supporting the energy supply and carbon utilization required for the reduction of fluorescent AGEs.

To further evaluate the carbohydrate utilization potential of HNGD-YC1, CAZyme annotation was performed. As presented in Figure 4E, a total of 133 CAZyme genes were identified in the genome, among which glycoside hydrolases (GH, 58 genes) and glycosyl transferases (GT, 33 genes) were the most abundant families, followed by carbohydrate esterases (CE, 16 genes), carbohydrate-binding modules (CBM, 16 genes), auxiliary activities (AA, 9 genes), and polysaccharide lyases (PL, 1 gene). The predominance of GH and GT genes suggests that HNGD-YC1 possesses an efficient carbohydrate metabolism system, supporting its fermentation potential.

Based on the integrated functional annotation results from eggNOG, GO, KEGG, and CAZyme databases, three candidate genes potentially involved in the reduction of fluorescent AGEs were identified (Table S1). GE000010 encodes a protease (eggNOG category [O]) capable of hydrolyzing peptide bonds and disrupting protein–protein crosslinks, which may facilitate the depolymerization of protein aggregates in AGEs. GE000160 encodes a glycoside hydrolase (GH family) that can catalyze the hydrolysis of glycosidic bonds within the glycan moieties of glycoproteins. GE000732 encodes a carbohydrate esterase (CE1 family) that may cleave ester bonds between protein and sugar components. The combined actions of these three candidate enzymes suggest a putative multi-enzymatic mechanism associated with the reduction of fluorescent AGEs. However, these genes are only candidates, and their involvement in the reduction of fluorescent AGEs remains to be experimentally validated.

To evaluate the genomic safety profile of HNGD-YC1, antibiotic resistance genes and potential virulence determinants were screened. Four antibiotic resistance-associated genes were detected, with sequence homologies to known resistance genes ranging from 29.49% to 48.23%, all below the 80% threshold considered concerning for horizontal gene transfer (Table S2). Furthermore, screening against the PHI-base identified only four genes with limited homology to known pathogenicity-related sequences, with sequence identities ranging from 24.84% to 48.46% (Table S3). These genomic assessments suggest a favorable safety profile, as HNGD-YC1 does not carry functional acquired antibiotic resistance genes or virulence factors, further supporting its potential as a food-grade microorganism candidate.

### 3.5. Reduction Effects of Strain HNGD-YC1 on Protein Oxidation Products in Spray-Dried Milk Powder

The efficacy of strain HNGD-YC1 in reducing fluorescent AGEs and other protein oxidation products was evaluated using spray-dried milk powder as a model food matrix. Pasteurized milk powder (freeze-dried) served as the low-oxidation control, while spray-dried milk powder represented the high-oxidation group. As shown in Figure 5A–G, compared with the control group, spray-dried milk powder exhibited significantly elevated levels of fluorescent AGEs, carbonyls, Schiff bases, kynurenine, and N′-formylkynurenine (*p* < 0.05), accompanied by marked reductions in free thiols and free amino groups (*p* < 0.05), confirming that the spray-drying process induced substantial protein oxidation. Following fermentation with HNGD-YC1 for 48 h, the fluorescent AGEs content in the oxidized milk powder decreased by 68.70%, while the levels of carbonyls, Schiff bases, kynurenine, and N′-formylkynurenine were also significantly reduced (*p* < 0.05). Concurrently, the contents of free thiols and free amino groups were significantly increased compared to the unfermented oxidized group (*p* < 0.05). These results demonstrate that HNGD-YC1 fermentation effectively reduces fluorescent AGEs and multiple other protein oxidation products in spray-dried milk powder.

The structural changes of milk powder proteins were examined using CLSM and FTIR spectroscopy. CLSM images of FITC-labeled proteins are presented in Figure 5H–J. The control group (Figure 5H) exhibited a homogeneous distribution of fine particles with consistent fluorescence intensity, indicating intact protein conformation. In the oxidized group (Figure 5I), distinct aggregated patches with intensified fluorescence emerged, characterized by crosslinked and irregular clusters, features typical of oxidation-induced protein aggregation. After fermentation (Figure 5J), these aggregates were largely dissipated, and the protein particles returned to a dispersed state, suggesting that the strain effectively disrupted oxidation-mediated crosslinks. The secondary structure changes of milk proteins were further analyzed by FTIR spectroscopy (Table S4). The control group showed an α-helix content of 21.63% and a β-sheet content of 32.87%, reflecting a well-preserved ordered structure. Spray-drying resulted in a decrease in α-helix and β-turn contents, along with an increase in β-sheet and random coil contents. After fermentation, the α-helix and β-turn contents partially recovered toward control levels, while the β-sheet content decreased, indicating that HNGD-YC1 fermentation attenuated the oxidation-associated structural alterations in milk proteins.

### 3.6. Reduction Effects of Strain HNGD-YC1 on Protein Oxidation Products in Roasted Pork Powder

To further validate the efficacy of strain HNGD-YC1 in a different food matrix, roasted pork powder was employed as a second model system. Sous-vide cooked pork powder served as the low-oxidation control, while oven-roasted pork powder (180 °C, 30 min) represented the high-oxidation group. As shown in Figure 6A–G, roasting led to significant increases in fluorescent AGEs, carbonyls, Schiff bases, kynurenine, and N′-formylkynurenine levels compared to the control (*p* < 0.05), coupled with substantial decreases in free thiols and free amino groups (*p* < 0.05), confirming that high-temperature roasting triggered severe protein oxidation. Following fermentation with HNGD-YC1 for 48 h, the fluorescent AGEs content in the oxidized pork powder decreased by 56.39% (Figure 6A), while the levels of carbonyls, Schiff bases, kynurenine, and N′-formylkynurenine were also significantly reduced (*p* < 0.05). Concurrently, the contents of free thiols and free amino groups were significantly higher than those in the unfermented oxidized group (*p* < 0.05), indicating that HNGD-YC1 fermentation attenuated fluorescent AGEs and multiple other oxidative modifications in roasted pork powder.

The structural alterations of pork proteins were investigated through CLSM and FTIR spectroscopy. CLSM images of FITC-labeled proteins are presented in Figure 6H–J. The control group (Figure 6H) displayed predominantly small, fragmented particles evenly distributed with uniform fluorescence, reflecting the intact state of myofibrillar proteins. In the roasted pork powder (Figure 6I), abundant elongated, dense myofibrillar structures with sharp outlines and locally augmented fluorescence were observed, indicating heat-induced crosslinking that rendered the proteins more rigid and resistant to homogenization. Following fermentation with HNGD-YC1 (Figure 6J), these elongated myofibrillar features largely vanished, and the proteins appeared as shorter, thicker, regularly shaped particles with enhanced overall fluorescence, suggesting effective alleviation of protein aggregation and crosslinking. The secondary structure changes of pork proteins were further analyzed by FTIR spectroscopy (Table S5). The control group exhibited an α-helix content of 23.82% and a β-sheet content of 35.08%, indicative of well-maintained ordered structures. High-temperature roasting caused a marked reduction in α-helix content and a sharp rise in β-sheet content, along with increases in random coil and decreases in β-turn content. After fermentation, the α-helix and β-turn contents partially recovered toward control levels, while the β-sheet and random coil contents decreased, indicating that HNGD-YC1 fermentation mitigated the oxidation-associated alterations in pork protein secondary structures induced by high-temperature roasting.

## 4. Discussion

The screening strategy employed in this study, using AGEs as the primary nitrogen source in a selective medium, was designed to enrich LAB capable of reducing fluorescent AGEs. Among the 1213 presumptive LAB isolates screened, only three strains (approximately 0.25%) exhibited AGEs reduction rates exceeding 50%, a finding consistent with other functional screening studies targeting specific foodborne substrates, in which high-efficiency strains also constituted a minor proportion of the isolated libraries [30,32]. The two-step screening strategy, comprising primary screening in AGEs selective medium followed by secondary verification in MRS broth supplemented with AGEs, confirmed the robustness of the candidate strains under nutritionally complex conditions. Strain HNGD-YC1 maintained a reduction rate of 52.00% in MRS medium, significantly higher than HNGD-KL5 (43.67%) and HNGD-WY4 (34.30%), indicating that its AGEs-reducing activity is constitutively expressed and not suppressed by competing nutrients, a trait particularly advantageous for practical food applications. This approach differs fundamentally from most previous investigations, which have primarily focused on inhibiting AGEs formation or reducing their intestinal absorption rather than reducing already formed AGEs in food matrices [33,53]. The identity of strain HNGD-YC1 was confirmed as *L. plantarum* through 16S rDNA sequencing, phylogenetic analysis, and whole-genome ANI analysis. This species is one of the most extensively studied probiotic LAB, renowned for its environmental adaptability, metabolic versatility, and well-established safety record [26,41].

The in vitro probiotic-related properties of strain HNGD-YC1 were systematically evaluated through its tolerance to simulated gastrointestinal conditions and adhesive properties. Strain HNGD-YC1 exhibited robust survival at pH 2.0 and 3.0, as well as in the presence of 0.15% and 0.3% bile salts, with survival rates comparable to or exceeding those reported for other *L. plantarum* strains [54,55]. Beyond survival, adhesion to the intestinal epithelium, an important property of potential probiotics, was indirectly estimated by cell surface hydrophobicity and auto-aggregation. HNGD-YC1 exhibited the highest hydrophobicity (95.99%) and auto-aggregation capacity (38.04%) among the three candidates, values notably higher than many other *L. plantarum* strains reported in the literature [54,55,56]. The combination of robust stress tolerance and superior adhesive properties supports the adhesion potential of HNGD-YC1 in vitro. The safety of HNGD-YC1 was evaluated through both phenotypic and genomic approaches. Phenotypically, it exhibited no hemolytic activity (γ-hemolysis), indicating the absence of hemolysin production. Genomically, whole-genome sequencing revealed no functional acquired antibiotic resistance genes (homology < 80% to known resistance genes) or virulence determinants, consistent with safety assessments of other *L. plantarum* strains [57,58]. These analyses suggest a favorable safety profile, but do not confer GRAS status or replace strain-specific safety assessment. The four antibiotic resistance-associated sequences showed only 29.49–48.23% identity to known resistance genes and were classified as Loose hits. No mobile genetic elements, such as plasmids, transposons, or integrons, were found in close proximity to these sequences, suggesting a low potential for horizontal gene transfer. Similarly, the four PHI-base hits showed only 24.84–48.46% identity to known pathogenicity-related sequences, further supporting a low virulence potential.

The genome of strain HNGD-YC1 harbors a rich repertoire of genes associated with carbohydrate transport and metabolism, energy production, and protein turnover, which collectively constitute a potential genetic foundation for the metabolic utilization of glycoprotein complexes such as AGEs. Notably, three candidate genes were identified as potentially involved in the reduction of fluorescent AGEs, each targeting a distinct structural component of these complex glycation products. GE000010 encodes a protease capable of hydrolyzing peptide bonds and disrupting protein–protein crosslinks, which may facilitate the depolymerization of protein aggregates in AGEs [59]. GE000160 encodes a glycoside hydrolase (GH family) that can catalyze the hydrolysis of glycosidic bonds within glycan moieties of glycoproteins [60,61]. GE000732 encodes a carbohydrate esterase (CE1 family) that may cleave ester bonds between protein and sugar components [62,63]. The combined actions of these three candidate enzymes, targeting peptide bond hydrolysis, glycosidic bond cleavage, and ester bond breakage, suggest a putative multi-enzymatic mechanism involved in the reduction of fluorescent AGEs. This putative mechanism is analogous to the three-step cooperative degradation model observed in microbial breakdown of recalcitrant organic compounds, involving extracellular enzymatic depolymerization, transmembrane transport, and intracellular metabolism [64]. The predominance of GH and GT genes in CAZyme annotation further supports the efficient hydrolysis of glycosidic bonds, consistent with the carbohydrate utilization versatility of *L. plantarum* strains. However, this putative mechanism is currently based solely on genomic evidence; functional expression of the corresponding enzymes and their direct involvement in the reduction of fluorescent AGEs require experimental validation through transcriptomic, proteomic, or gene knockout approaches. Nevertheless, the genomic evidence presented here offers the first genetic clues for the direct reduction of pre-formed fluorescent AGEs by a *L. plantarum* strain, providing a foundation for understanding the molecular basis of LAB-mediated reduction of fluorescent AGEs.

Strain HNGD-YC1 effectively reduced fluorescent AGEs and other protein oxidation products in both spray-dried milk powder and roasted pork powder, with reduction rates reaching 68.70% and 56.39%, respectively. To the best of our knowledge, this is the first report of a *L. plantarum* strain capable of directly reducing pre-formed fluorescent AGEs in complex food matrices, as previous studies have predominantly focused on inhibiting AGEs formation or reducing their intestinal absorption [33,53]. The higher reduction efficiency observed in milk powder compared to pork powder may be attributed to differences in protein types, oxidation conditions, and matrix structure affecting bacterial accessibility [52]. Notably, HNGD-YC1 fermentation also decreased the levels of multiple other protein oxidation products and increased free thiols and free amino groups in both matrices. These changes may be partly explained by the putative multi-enzymatic mechanism suggested by genomic analysis. However, acidification and general proteolytic activity during fermentation may also contribute to the reductions in detectable fluorescent AGEs and other oxidation markers, and the increases in free thiols and amino groups may partly result from proteolytic release and structural changes in the protein matrix. Therefore, the observed reductions are reported as reductions in detectable fluorescent AGEs rather than as definitive evidence of direct microbial degradation. Beyond biochemical evidence, microstructural and conformational analyses confirmed that fermentation alleviated protein aggregation and partially recovered the secondary structure parameters, with α-helix content increasing and β-sheet content decreasing toward the levels of unoxidized controls (Tables S4 and S5). Similar structural modifications of food matrices by microbial fermentation have been reported, including changes in dietary fiber properties and protein conformation [65]. These findings indicate that HNGD-YC1 fermentation not only reduces oxidation products but also attenuates the oxidation-induced alterations in protein secondary structure.

This study has several limitations. Specific AGEs such as CML and CEL were not quantified, and the reduction in fluorescent AGEs could not be fully distinguished from adsorption, nonspecific binding, or extracellular enzymatic activity, because heat-inactivated cell, cell-free supernatant, and biomass controls were not included. In addition, an oxidized matrix incubated for 48 h without bacteria and an acidified control were not included, so the contributions of time/temperature and acidification could not be fully separated from microbial activity. The putative mechanism is based solely on genomic annotation and lacks functional validation, and the products formed after the reduction of fluorescent AGEs were not analyzed, so it cannot be concluded that the reduced fluorescent AGEs were detoxified or converted into harmless metabolites. Furthermore, no in vivo studies were conducted, phenotypic antibiotic susceptibility testing was not performed, and the safety and stability of the strain require further confirmation before food applications. Future studies integrating LC-MS/MS quantification of specific AGEs, functional gene validation, appropriate control experiments, product analysis, phenotypic antibiotic susceptibility testing, and in vivo trials will be necessary to confirm the mechanism, safety, and potential applications of HNGD-YC1.

## 5. Conclusions

In this study, a food-grade lactic acid bacterium, *Lactiplantibacillus plantarum* HNGD-YC1, capable of efficiently reducing fluorescent AGEs, was isolated from naturally fermented foods using a targeted screening strategy. The strain exhibited favorable in vitro probiotic-related properties and a safety profile based on genomic and hemolytic analyses, supporting its potential for use as a food-grade microorganism. Whole-genome sequencing revealed candidate genes (GE000010, GE000160, and GE000732) encoding proteases, glycoside hydrolases, and carbohydrate esterases, suggesting a putative multi-enzymatic mechanism associated with the reduction of fluorescent AGEs. In food matrix applications, HNGD-YC1 fermentation reduced fluorescent AGEs by 68.70% in spray-dried milk powder and 56.39% in roasted pork powder, while also reducing multiple other protein oxidation products and partially recovering protein secondary structure parameters. These findings establish HNGD-YC1 as a potential probiotic candidate for reducing fluorescent AGEs and other oxidation-associated markers in food matrices, offering a potential strategy for developing fermented products with reduced fluorescent AGEs content.

## Figures and Tables

**Figure 1:**
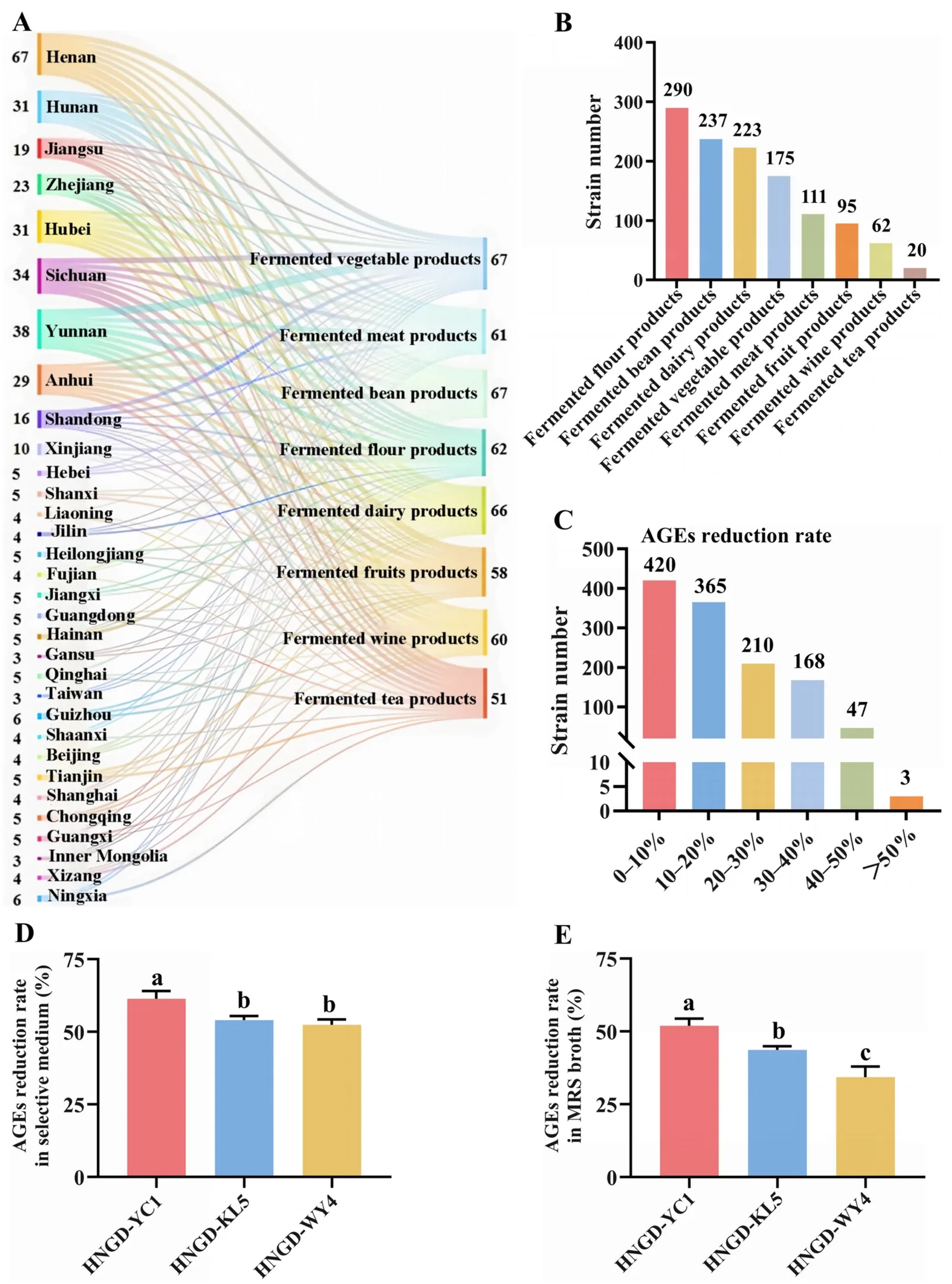
The origin of fermented foods and screening of LAB for reduction of fluorescent Advanced glycation end products (AGEs). (**A**) Geographic distribution and categories of fermented food samples. (**B**) Number of presumptive lactic acid bacteria isolates obtained from each fermented food category. (**C**) Distribution of 1213 isolates by reduction rates in AGEs selective medium (*x*-axis: reduction rate intervals; *y*-axis: number of isolates). Reduction rates of three candidate strains in AGEs selective medium (**D**) and MRS broth (**E**). Data are mean ± SEM of three independent biological replicates (*n* = 3). Different lowercase letters indicate significant differences among strains (*p* < 0.05).

**Figure 2:**
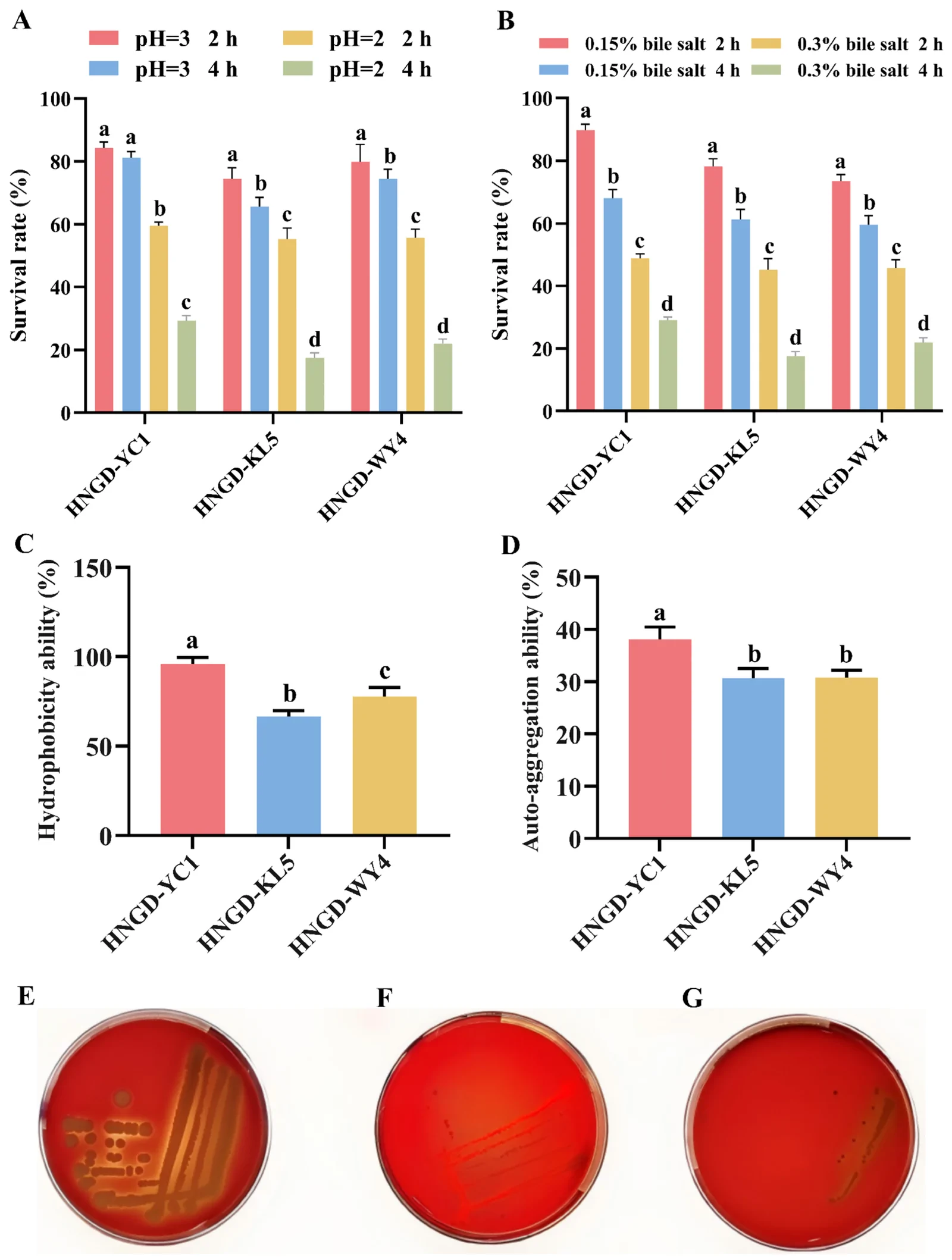
In vitro probiotic-related properties and safety evaluation of the selected strain. (**A**) Survival rates under acidic conditions (pH 2.0 and 3.0). (**B**) Survival rates under bile salt conditions (0.15% and 0.3%). (**C**) Hydrophobicity ability of strains. (**D**) Auto-aggregation ability of strains. (**E**–**G**) Hemolytic activity on Columbia blood agar plates: (**E**) *Staphylococcus aureus* ATCC 25923 (positive control, β-hemolysis, clear zones around colonies); (**F**) *Lactiplantibacillus plantarum* ATCC 14917 (negative control, γ-hemolysis, no clear zones); (**G**) Strain HNGD-YC1 (γ-hemolysis, no clear zones). The red background is the blood agar medium, and clear zones around colonies indicate β-hemolysis. Data are mean ± SEM of three independent biological replicates (*n* = 3). Different lowercase letters indicate significant differences among strains (*p* < 0.05).

**Figure 3:**
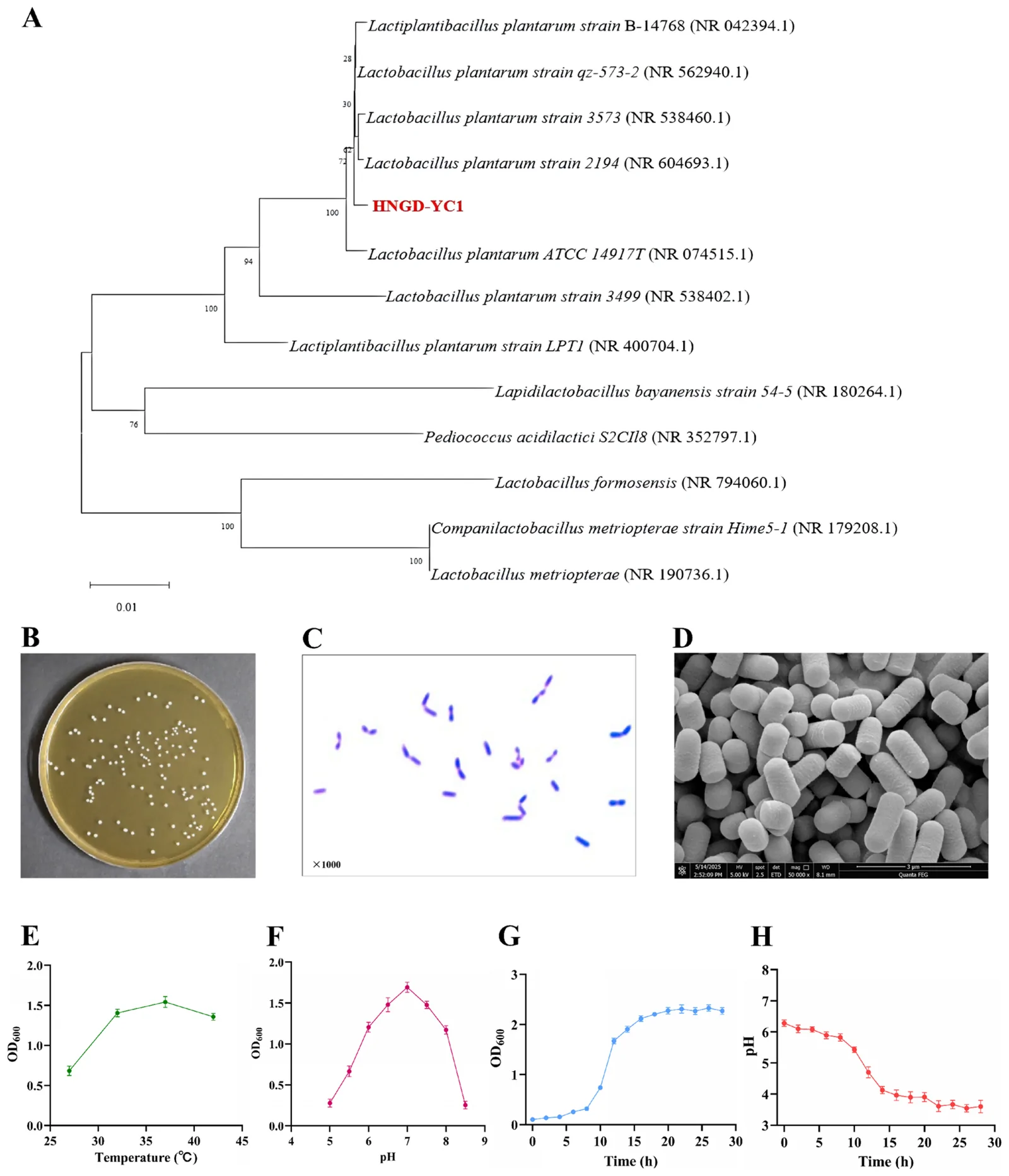
Identification, morphological, and growth characteristics of strain HNGD-YC1. (**A**) Phylogenetic tree based on 16S rDNA gene sequences. (**B**) Colony morphology. (**C**) Gram staining (1000× magnification). (**D**) Scanning electron microscopy image (50,000× magnification). (**E**) Optimal growth temperature. (**F**) Optimal pH. (**G**) Growth curve. (**H**) Acid production curve. Data are mean ± SEM of three independent biological replicates (*n* = 3).

**Figure 4:**
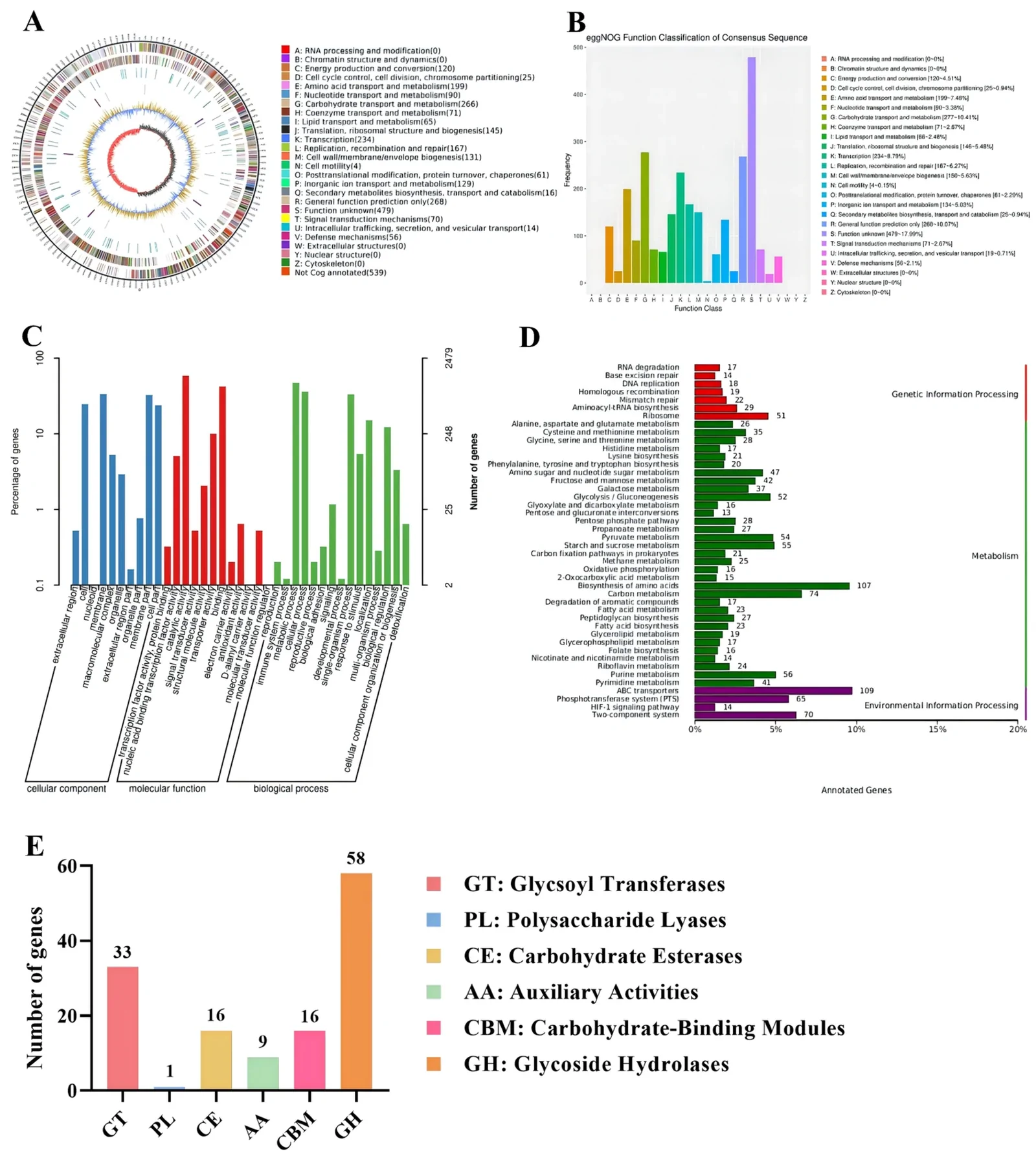
Whole-genome sequencing and functional annotation of strain HNGD-YC1. (**A**) Circular genome map of strain HNGD-YC1. (**B**) eggNOG functional classification. (**C**) GO functional classification. (**D**) KEGG pathway enrichment analysis. (**E**) CAZyme family gene distribution.

**Figure 5:**
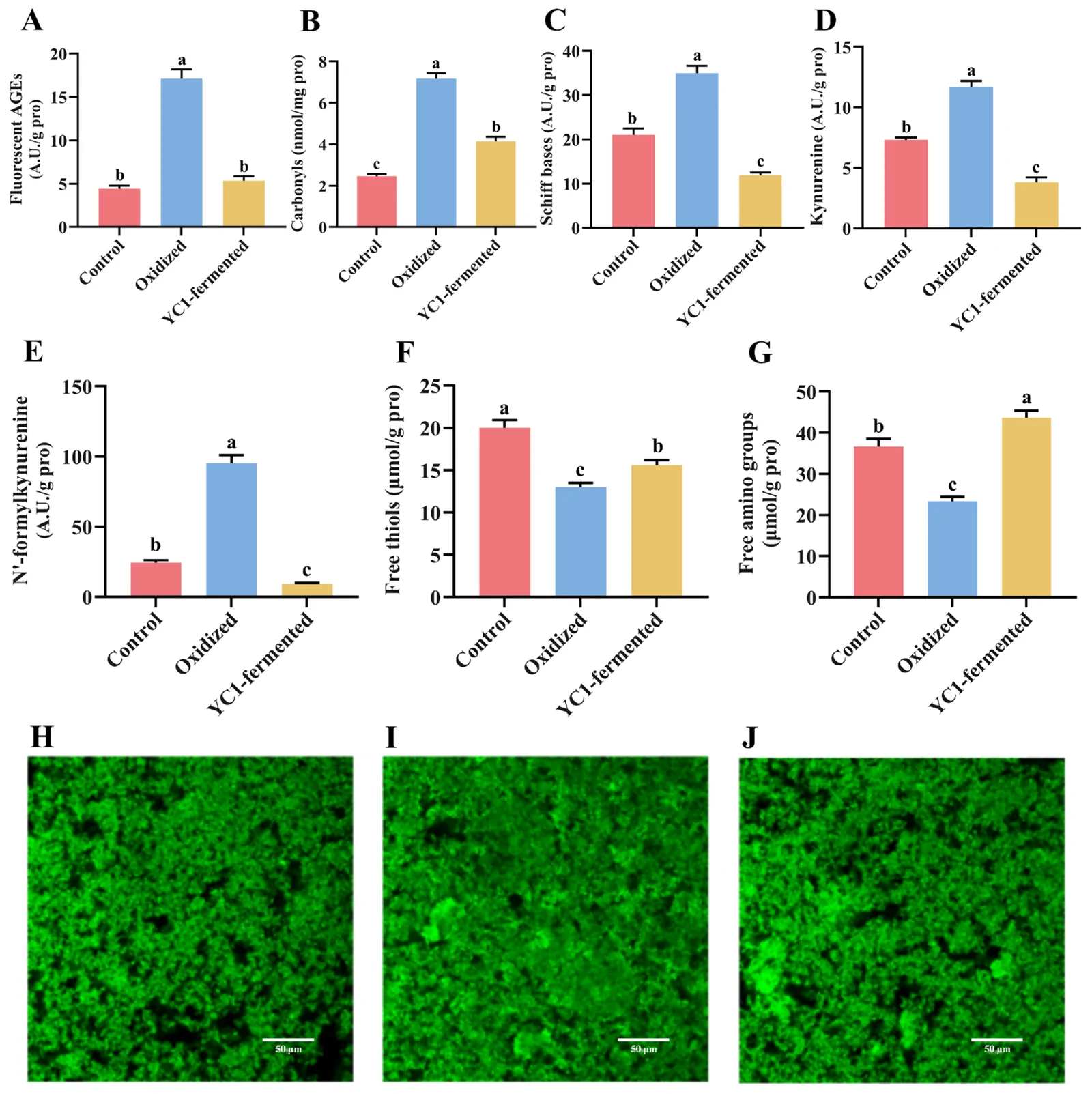
Reduction effects of strain HNGD-YC1 on protein oxidation products in spray-dried milk powder. Levels of (**A**) Fluorescent AGEs, (**B**) carbonyls, (**C**) Schiff bases, (**D**) kynurenine, (**E**) N′-formylkynurenine, (**F**) free thiols, and (**G**) free amino groups. CLSM images of milk powder proteins in (**H**) control, (**I**) oxidized, and (**J**) YC1-fermented groups. Scale bar = 20 μm. Data are mean ± SEM of three independent biological replicates (*n* = 3). Different lowercase letters indicate significant differences among groups (*p* < 0.05).

**Figure 6:**
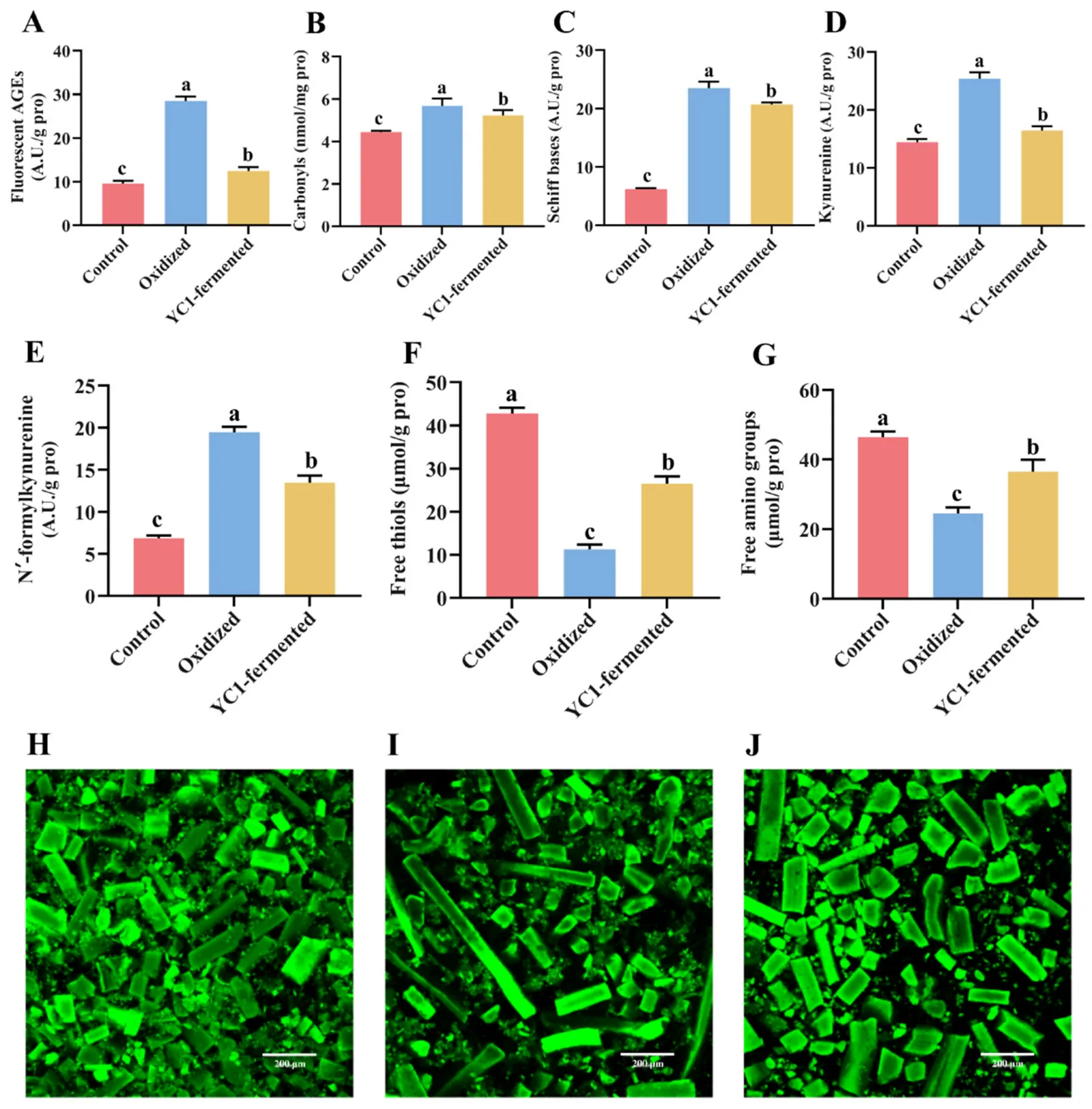
Reduction effects of strain HNGD-YC1 on protein oxidation products in roasted pork powder. Levels of (**A**) Fluorescent AGEs, (**B**) carbonyls, (**C**) Schiff bases, (**D**) kynurenine, (**E**) N′-formylkynurenine, (**F**) free thiols, and (**G**) free amino groups. CLSM images of pork powder proteins in (**H**) control, (**I**) oxidized, and (**J**) YC1-fermented groups. Scale bar = 200 μm. Data are mean ± SEM of three independent biological replicates (*n* = 3). Different lowercase letters indicate significant differences among groups (*p* < 0.05).

## Data Availability

The original contributions presented in this study are included in the article/Supplementary Materials. The genome sequence of strain HNGD-YC1 has been deposited in NCBI under BioProject PRJNA1530792 and BioSample SAMN63202719. Further inquiries can be directed to the corresponding author.

## Supplementary Materials

The following supporting information can be downloaded at: https://www.mdpi.com/article/10.3390/foods15193403/s1. Table S1: Putative genes in strain HNGD-YC1 involved in the reduction of fluorescent AGEs; Table S2: Antibiotic resistance genes identified in the genome of strain HNGD-YC1; Table S3: PHI-base annotation statistics for potential pathogenicity-related genes in strain HNGD-YC1; Table S4: Effects of HNGD-YC1 fermentation on the secondary structure of milk powder proteins; Table S5: Effects of HNGD-YC1 fermentation on the secondary structure of pork powder proteins.

## Abbreviations

The following abbreviations are used in this manuscript:

AGEs	Advanced glycation end products
LAB	Lactic acid bacteria
GRAS	Generally recognized as safe
CML	Carboxymethyl lysine
CEL	Carboxyethyl lysine
PBS	Phosphate-buffered saline
MRS	Man–Rogosa–Sharpe
OD_600_	optical density at 600 nm
eggNOG	Evolutionary Eenealogy of Genes: Non-supervised Orthologous Groups
GO	Gene Ontology
KEGG	Kyoto Encyclopedia of Genes and Genomes
CAZymes	Carbohydrate-active enzymes
PHI-base	Pathogen–Host Interaction database
CLSM	Confocal laser scanning microscopy
FITC	Fluorescein isothiocyanate
FTIR	Fourier-transform infrared
SEM	standard error of the mean
ANOVA	analysis of variance
GH	Glycoside hydrolases
GT	Glycosyl transferases
CE	Carbohydrate esterases
CBM	Carbohydrate-binding modules
AA	Auxiliary activities
PL	Polysaccharide lyases
