# Supplementary material for: Screening and Evaluation of Lactiplantibacillus plantarum HNGD-YC1 for Mitigation of Advanced Glycation End Products in Food Matrices
Source: Foods. 2026 Sep 24;15(19):3403. doi: 10.3390/foods15193403 (PMC13664014; doi:10.3390/foods15193403)
Supplement: Supplementary file 1 [file foods-15-03403-s001.zip › foods-4529341-supplementary.pdf]

Supplementary Materials

Screening and Evaluation of *Lactiplantibacillus plantarum* HNGD-YC1 for Mitigation of Advanced Glycation End Products in Food Matrices

Yueting Ge[1,2,3], Kaile Chen[4], Zhenguo Tian[4], Chengjia Gu[1,2], Boyang Kou[1,2], Guowei Le[5], Yuhui Yang[4*]

[1] Henan Key Laboratory of Tea Plant Biology, College of Tea and Food Science, Xinyang Normal University, Xinyang 464000, China

[2] Dabie Mountain Laboratory, Xinyang 464000, China

[3] Henan International Joint Laboratory of Tea-oil tree Biology and High-Value Utilization, College of Tea and Food Science, Xinyang Normal University, Xinyang 464000, China

[4] College of Food Science and Engineering, Henan University of Technology, Zhengzhou 450001, China

[5] State Key Laboratory of Food Science and Technology, School of Food Science and Technology, Jiangnan University, Wuxi 214122, China

* Correspondence: Yuhui Yang, E-mail: yangyuhui1992@126.com

College of Food Science and Engineering, Henan University of Technology

100 Lotus Street, Hi-tech Development District, Zhengzhou 450001, China.

Tel.: +86-371-67758022; Fax: +86-371-67758021

Supplemental Tables

Table S1. Putative genes in strain HNGD-YC1 involved in the reduction of fluorescent AGEs.

Gene ID	Database/Category	Putative function
GE000010	eggNOG / [O] Post-translational modification, protein turnover	Encodes a protease that hydrolyzes peptide bonds and breaks protein–protein crosslinks, potentially depolymerizing protein aggregates induced by oxidation [1]
GE000160	KEGG / GH family (Glycosaminoglycan degradation pathway)	Encodes a glycosaminoglycan hydrolase that catalyzes glycosidic bond hydrolysis, cleaving glycan side chains in glycoproteins [2,3]
GE000732	CAZyme / CE1 (Carbohydrate esterase)	Encodes a carbohydrate esterase that catalyzes ester bond hydrolysis, breaking ester crosslinks between proteins and glycans [4,5]

Note: These genes were identified based on genomic annotation and are predicted to be involved in the reduction of fluorescent AGEs.

Table S2. Antibiotic resistance genes identified in the genome of strain HNGD-YC1.

Gene ID	Resistance type	Resistance mechanism	Identity (%)	Coverage (%)
GE000033	Macrolide antibiotic, penam	Antibiotic efflux	48.23	96.17
GE000034	Disinfecting agents and antiseptics	Antibiotic efflux	35.84	36.22
GE000046	Fusidic acid	Antibiotic target protection	41.09	93.02
GE000068	Tetracycline antibiotic	Antibiotic target protection	29.49	88.84

Note: The sequence homologies to known resistance genes were all below the 80% threshold, suggesting a low risk of horizontal gene transfer.

Table S3. PHI-base annotation statistics for potential pathogenicity-related genes in strain HNGD-YC1.

Gene ID	Pathogen species	Identity (%)	Coverage (%)
GE000006	*Burkholderia glumae*	48.46	93.65
GE000008	*Xanthomonas oryzae*	30.93	47.00
GE000010	*Streptococcus pneumoniae*	32.83	98.21
GE000018	*Fusarium oxysporum*	24.84	67.03

Note: The sequence identities were well below the threshold for virulence determinants, indicating no significant pathogenicity-related genes.

Table S4. Effects of HNGD-YC1 fermentation on the secondary structure of milk powder proteins.

	α-Helix (%)	β-Sheet (%)	β-Turn (%)	Random coil (%)
Control	21.63 ± 2.25^{a}	32.87 ± 3.38^{c}	27.14 ± 2.78^{a}	18.36 ± 1.87^{a}
Oxidized	18.76 ± 1.95^{b}	42.34 ± 3.83^{a}	16.68 ± 2.17^{c}	22.22 ± 2.21^{a}
YC1-fermented	20.82 ± 2.14^{ab}	35.08 ± 3.55^{b}	23.73 ± 2.43^{b}	20.37 ± 2.02^{a}

Note: Control: Pasteurized milk powder (low-oxidation); Oxidized: Spray-dried milk powder; YC1-fermented: Spray-dried milk powder fermented with HNGD-YC1. Values are expressed as mean ± SEM of three independent biological replicates (n = 3). Different superscript letters within the same column indicate significant differences among groups ($p < 0.05$).

Table S5. Effects of HNGD-YC1 fermentation on the secondary structure of pork powder proteins.

	α-Helix (%)	β-Sheet (%)	β-Turn (%)	Random coil (%)
Control	23.82 ± 2.57^{a}	35.08 ± 2.89^{b}	30.24 ± 3.66^{a}	10.86 ± 1.13^{c}
Oxidized	11.47 ± 1.25^{c}	48.32 ± 4.85^{a}	21.73 ± 2.24^{b}	18.48 ± 1.94^{a}
YC1-fermented	15.23 ± 1.53^{b}	37.07 ± 3.51^{b}	32.42 ± 3.27^{a}	15.28 ± 1.76^{b}

Note: Control: Sous-vide cooked pork powder (low-oxidation); Oxidized: roasted pork powder; YC1-fermented: roasted pork powder fermented with HNGD-YC1. Values are expressed as mean ± SEM of three independent biological replicates (n = 3). Different superscript letters within the same column indicate significant differences among groups ($p < 0.05$).

References

1. Cohen-Or, I.; Katz, C.; Ron, E.Z. Metabolism of AGEs–Bacterial AGEs are degraded by metallo-proteases. *PLoS ONE*. **2013**, *8*, e74970.

2. Dong, J.; Cui, Y.; Qu, X. Metabolism mechanism of glycosaminoglycans by the gut microbiota: Bacteroides and lactic acid bacteria: A review. *Carbohydr. Polym*. **2024**, *332*, 121905.

3. Cobucci-Ponzano, B.; Conte, F.; Strazzulli, A.; Capasso, C.; Fiume, I.; Pocsfalvi, G.; Rossi, M.; Moracci, M. The molecular characterization of a novel GH38 α-mannosidase from the crenarchaeon Sulfolobus solfataricus revealed its ability in de-mannosylating glycoproteins. *Biochimie*. **2010**, *92*, 1895–1907.

4. Zhang, H.; Wen, B.; Liu, Y.; Wang, Z.; Xu, Y.; Zhang, J. A reverse catalytic triad Asp containing loop shaping a wide substrate binding pocket of a feruloyl esterase from Lactobacillus plantarum. *Int. J. Biol. Macromol*. **2021**, *184*, 92–100.

5. Vaaje-Kolstad, G.; Westereng, B.; Horn, S.J.; Liu, Z.; Zhai, H.; Sørlie, M.; Eijsink, V.G.H. An oxidative enzyme boosting the enzymatic conversion of recalcitrant polysaccharides. *Science*. **2010**, *330*, 219–222.
